# Bleeding management after implementation of the Hemorrhage Code (Code H) at the Hospital Israelita Albert Einstein, São Paulo, Brazil

**DOI:** 10.31744/einstein_journal/2020AO5032

**Published:** 2020-08-25

**Authors:** Michele Jaures, Neila Maria Marques Negrini Pigatti, Roseny dos Reis Rodrigues, Fernanda Paulino Fernandes, João Carlos de Campos Guerra

**Affiliations:** 1 Hospital Israelita Albert Einstein São Paulo SP Brazil Hospital Israelita Albert Einstein, São Paulo, SP, Brazil.

**Keywords:** Shock, hemorrhagic, Hemorrhage, Blood transfusion, Blood coagulation disorders

## Abstract

**Objective:**

To describe the implementation of a care protocol based on rapid response teams, for management and resolution of bleeding.

**Methods:**

A hospital protocol called Hemorrhage Code (Code H) was devised and developed. In a flow line, a multidisciplinary team provides comprehensive, fast and effective care to the patient with a severe hemorrhagic condition. In another flow line, professionals based at the hospital pharmacy focus on identifying patients at risk of bleeding, to avoid this event. Several hospital professionals and sectors were trained, each with specific roles, ensuring full support to the medical and nursing staffs.

**Results:**

After implementing this protocol, we were able to significantly reduce the number of catastrophic events related to failure in bleeding management.

**Conclusion:**

Code H is an example of a value-based medicine and precision medicine project by delivering comprehensive and multidisciplinary care, in addition to point-of-care testing introduced in clinical practice, optimizing patient safety and care practices at the hospital. Furthermore, it will be possible to minimize the risk of lawsuits for the hospital and physicians, as well as rationalizing resources with benefits for administrators and payers.

## INTRODUCTION

Severe hemorrhage is an important cause of mortality and morbidity in several clinical settings, including trauma, surgery and obstetrics.^(1-7)^ The estimated total number of deaths associated with hemorrhage and hemorrhagic shock is 1.9 million per year, worldwide.^(8)^ Hemorrhagic shock is characterized by severe blood loss, which leads to inadequate oxygen delivery at cell level and quickly results in death. The median time from onset to death is 2 hours.^(9)^

Non-fatal hemorrhagic events require additional therapies, prolonged hospital stay, use of hemostatic agents, and discontinuing antithrombotic agents. This interruption, in turn, can result in a negative outcome for patients at risk for thrombosis.^(1-6)^

**Figure 1:**
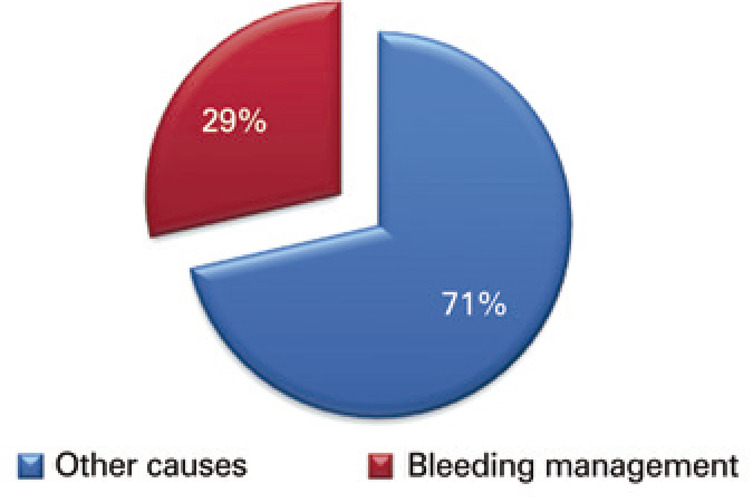
Catastrophic adverse events associated with inadequate bleeding management

At the *Hospital Israelita Albert Einstein (HIAE)*, from January 2013 to April 2016, the number of catastrophic adverse events related to failure in bleeding management was 29% (n=14) among the total catastrophic adverse events at the organization (n=49) (Figure 1).

The cause of a hemorrhage can be complex, and its management may be limited due to redundant diagnostic tools^(10)^ and clinical protocols. One of the key points of a successful treatment is the timing of the intervention. In addition to rapid interventions, a coordination between the different services in the hospital environment and a preventive bleeding management plan are essential.^(7)^

Several hemorrhage management approaches have been described in the last decades. The most recent ones recommend rapid control of bleeding, early management of the coagulation disorder, maintenance of adequate perfusion, and minimization of the inflammatory response.^(11)^

**Figure 2:**
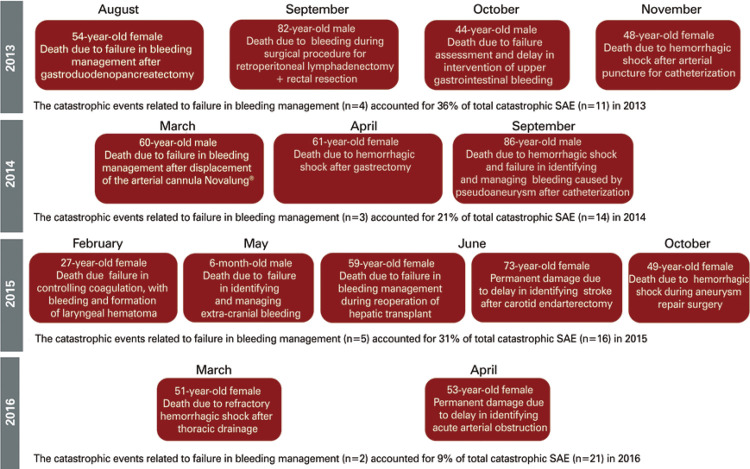
Description of catastrophic adverse events due to failure in bleeding management from January 2013 to April 2016 SAE: serious adverse event.

During the root cause analysis (RCA) of catastrophic adverse events at the HIAE (Figure 2), it was observed that the failures occurred due to lack of early recognition of bleeding, treatment failures, and the lack of logistical planning and communication between the hospital support areas involved in diagnosis and treatment of patients, resulting in delayed diagnosis and patient care.

In 2004, the Institute for Healthcare Improvement (IHI), through “The 100,000 Lives” campaign, recommended the deployment of rapid response teams (RRT) in hospitals, as one of six strategies to reduce the occurrence of unexpected deaths. Rapid response teams is a group of professionals with expertise in critical/intensive care, which should be quickly called at the patients’ bedside.

The RRT strategy raises the hypothesis that the implementation of a multidisciplinary protocol could avoid adverse events related to failures in management and recognition of a hemorrhagic shock.

## OBJECTIVE

To describe the implementation of a rapid response team health care protocol, for the management and resolution of bleeding conditions.

## METHODS

This was a retrospective experience report, on the actions taken for the implementation of the Hemorrhage Code, or Code H, a value-based medicine and precision medicine project, from May 2016 to June 2019. With this protocol, through comprehensive and multidisciplinary care, and point-of-care testing introduced in clinical practice, patient safety and health care practices have been improved.

**Figure 3:**
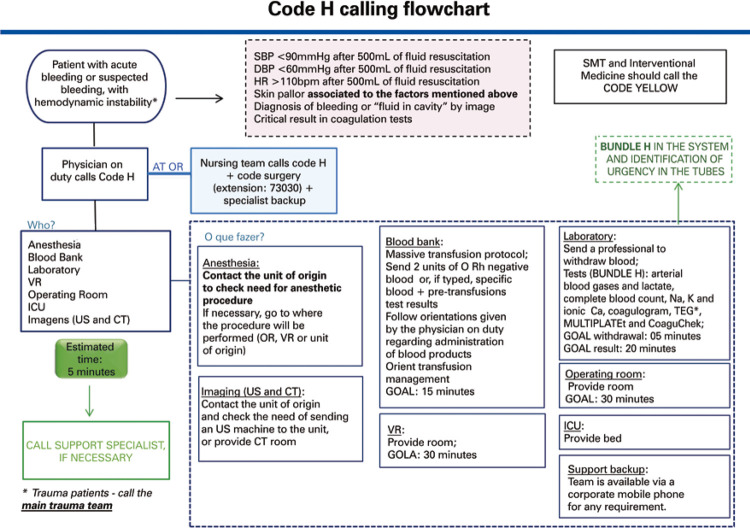
Hemorrhage Code (Code H) care flowchart * Trauma patient: the trauma team should be called. OR: operating room; SBP: systolic blood pressure; DBP: diastolic blood pressure; HR: heart rate; SMT: surgical medical team; VR: vascular radiology; ICU: intensive care unit; US: ultrasound; CT: computed tomography; Na: sodium; K: potassium; Ca: calcium; TEG: thromboelastogram.

The main objectives of Code H are early identification of signs and symptoms of bleeding, and the rapid implementation of treatment, through the emergency services of the hospital. This protocol requires a well-coordinated team from different departments of the hospital, such as the intensive care unit (ICU), anesthesia team, blood bank, clinical laboratory, diagnostic imaging services, vascular intervention team and operating room (Figure 3).

The parameters used to activate the Code H, the so-called “trigger points,” are described in table1.

**Table 1:** Trigger points

1. Systolic blood pressure ≤90mmHg
2. Mean diastolic blood pressure ≤60mmHg
3. Heart rate ≥110bpm
4. Skin and mucosal pallor associated with imaging tests that point to “active bleeding”
5. Hematoma or collection suggestive of bleeding in cavities
6. Critical results in clotting tests

Process and outcome indicators for actions before and after Code H were recorded between January 2013 and June 2019. The measurements and analyses focused on the following metrics: number of adverse events related to inadequate management of bleeding, and number of Code H activations, as of May 2016.

The ethical aspects of the report were respected, since this was a retrospective data survey in medical records, which did not interfere with the care received by the patients. The present study did not require the Informed Consent Form.

Following international bleeding management recommendations and through a multidisciplinary analysis of catastrophic adverse events, a plan of action was designed to ensure rapid bleeding control, early management of coagulation disorders, maintenance of adequate perfusion, and supply of adequate services in a timely manner, according to the causes of the bleeding.

Thus, as one or more trigger point criteria are identified, the Code H is activated by calling a specific phone number at the patient care location. Automatically, all related services are notified of the call (laboratory, blood bank, operating room, interventional radiology, imaging services, ICU, endoscopy, emergency room surgeon, transport service and the hospital anesthesia care team), with the response times recommended by the protocol (Figure 3).

From then on, a series of sequential and parallel actions are performed by different healthcare professionals (Figure 3):

- Nursing professionals initiate or maintain patient warming measures, interrupt medications that potentiate bleeding (such as antiplatelet, anticoagulant and thrombolytic drugs) and quantify the bleeding. This helps the medical decision-making process to implement blood transfusion protocols.
- Medical professionals are responsible for deciding whether or not to start transfusion of blood components immediately, and for making efforts to maintain normothermia and quickly regulate serum calcium levels and physiological blood pH. In addition, they identify whether the hemorrhagic shock has a surgical or coagulopathic etiology, in order to forward the patient to the correct site as soon as possible for a swift resolution of the bleeding.
- The laboratory staff ensures that sample collection is carried out within 5 minutes after the Code H activation, and that the results are released within the next 20 minutes. The Code H collection bundle includes a complete blood count with platelets, arterial blood gases and lactate levels, coagulogram with serum fibrinogen concentration, ionic calcium levels, CoaguChek for point-of-care international normalized ratio (INR) measurement at the bedside, thromboelastometry, and platelet function assessment tests.
- The blood bank staff is responsible for providing the transport of two units of group O Rh negative red blood cell concentrates within the first 20 minutes of the activation. If the physician responsible for the activation launches a massive transfusion protocol, the blood bank staff is responsible for maintaining the flow of blood components, as required by the circumstances of the patient’s condition.

The services for surgical or clinical resolution of bleeding are activated to provide a bed, according to the need indicated by the physician.

## RESULTS

The implementation of a multidisciplinary protocol required continuous training of all members involved. The training of the teams, the profile and the performance of the professionals were the main success factors for the positive result of Code H. These professionals demonstrated to be endowed with technical skills, systemic vision, situational awareness, and practical problem-solving abilities, for bleeding recognition and management. In the initial stage, approximately 530 employees were trained, which corresponded to more than 1,100 hours of training.

**Figure 4:**
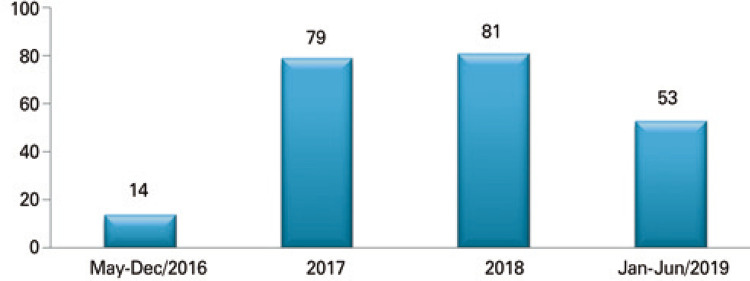
Number of Code H activations in the period from May 2016 to June 2019

From the implementation until June 2019, the Code H was activated 227 times (Figure 4). We observed an underutilization of the code at the beginning of its implementation, an excessive activation in the medium term, and a subsequent adaptation of the activation in the following periods.

One of the most important causes for not activating the Code H was based on the perception of the physician responsible for the patient, on his ability to manage the hemorrhagic condition without the need for other support teams. The continuous training and the unconditional support of the organization to train the teams were decisive for a change in culture, and an increase in the volume of activations.

The organization has been working with models of care using RRT since 2005 with great success, and the creation of the bleeding management code based on this structure brought familiarity to the teams and a quick dissemination of easiness of accessibility of services with the activation. Before the proposed model, the various needs, such as reservation of blood bags, operating rooms or exams, were carried out individually and not sequentially, causing delays in patient care.

**Figure 5:**
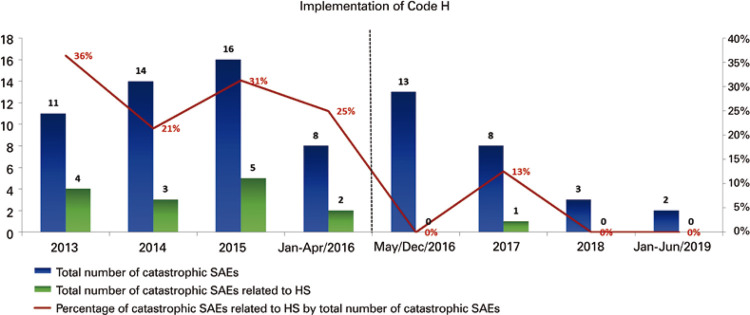
Evolution of total and hemorrhagic shock-related catastrophic adverse events before and after the implementation of the Hemorrhage Code (Code H) SAE: severe adverse event; HS: hemorrhagic shock.

After the implementation of the Code H, in May 2016, up to June 2019, there was a significant decrease in the number of catastrophic adverse events related to failures in bleeding management (only one case in 2017), as shown in figure 5.

## DISCUSSION

Hemorrhage as a result of surgical procedures or due to coagulation disorders occurs within the statistics of complications of some procedures.^(1-7)^ Interventions to control the bleeding and hemostatic resuscitation have proven to be useful in decreasing mortality from hemorrhagic injury.^(12)^

In the Stop The Bleed campaign the following elements were described as essential for the control of bleeding: evaluation of patients at risk of coagulopathic bleeding; immediate treatment of bleeding and coagulation disorder as soon as they develop; continuous observation of the response to interventions; and development of strategies to avoid secondary bleeding and coagulation disorders.^(13)^

Root cause analysis is one of the risk management tools widely used at the organization. In the present study, RCA was associated with the expertise of the RRT in the preparation of a multidisciplinary and sequenced protocol for bleeding management. Since its creation in the 2000s, RRTs have been implemented in different parts of the world and in diverse clinical cases, with much success.^(14)^

Code H also adds the following as important elements: the response time, the allocation of the patient in the correct place, and multidisciplinary and multisectoral coordination.

It is important to highlight a positive aspect involved in the formation of multidisciplinary teams arising from their training for the implementation of Code H. The information base and the guidelines provided to the teams brought greater involvement and perception of the signs of bleeding by the multidisciplinary teams. Significant effects of this training were demonstrated in the gradual increase in the number of activations, and in the resolution of bleeding conditions, providing a positive outcome for the patients. The lack of recognition of signs of bleeding is one of the weaknesses identified in care failures, and has been described in several studies in the literature as a key point in maintaining the life of the patients. A reproducible and sustainable training program was devised for the teams, to better disseminate knowledge on bleeding and train new employees, and also to strengthen the skills of the teams.

The number of catastrophic adverse events in the organization has been considerably reduced after the implementation of the protocol, confirming the importance of this type of initiative for the continuous improvement of health services.

There are certain limitations to this study, mainly in the financial cost of implementing the Code H. Additional studies are being carried out in order to assess the impact of this initiative on hospital management.

## CONCLUSION

The implementation of an emergency treatment protocol, with investments in medical and multidisciplinary staff training, reduced the number of bleeding-related adverse events in the organization. The systematization of the service reduced the failures related to the treatment of this complication, with an impact on the reduction of morbidity and mortality. Therefore, we concluded that the Code H contributes positively to the quality and safety of the care provided to patients with bleeding disorders.

The Code H is an example of a value-based medicine and precision medicine project, in which, through comprehensive and multidisciplinary care, and point-of-care tests introduced in clinical practice, we improved patient safety and care practice in our hospital. As additional results, this will probably minimize the risk of lawsuits against the hospital and the physicians, and will also streamline resources, with benefits for administrators and payers.

## References

[1] 1. Manoukian SV, Feit F, Mehran R, Voeltz MD, Ebrahimi R, Hamon M, et al. Impact of major bleeding on 30-day mortality and clinical outcomes in patients with acute coronary syndromes: an analysis from the ACUITY Trial. J Am Coll Cardiol. 2007;49(12):1362-8. 10.1016/j.jacc.2007.02.027 17394970

[2] 2. Mehran R, Pocock SJ, Stone GW, Clayton TC, Dangas GD, Feit F, et al. Associations of major bleeding and myocardial infarction with the incidence and timing of mortality in patients presenting with non-ST-elevation acute coronary syndromes: a risk model from the ACUITY trial. Eur Heart J. 2009;30(12):1457-66. 10.1093/eurheartj/ehp110 PMC2695951 19351691

[3] 3. Eikelboom JW, Mehta SR, Anand SS, Xie C, Fox KA, Yusuf S. Adverse impact of bleeding on prognosis in patients with acute coronary syndromes. Circulation. 2006;114(8):774-82. 10.1161/CIRCULATIONAHA.106.612812 16908769

[4] 4. Rao SV, O’Grady K, Pieper KS, Granger CB, Newby LK, Van de Werf F, et al. Impact of bleeding severity on clinical outcomes among patients with acute coronary syndromes. Am J Cardiol. 2005;96(9):1200-6. 10.1016/j.amjcard.2005.06.056 16253582

[5] 5. Segev A, Strauss BH, Tan M, Constance C, Langer A, Goodman SG; Canadian Acute Coronary Syndromes Registries Investigators. Predictors and 1-year outcome of major bleeding in patients with non-ST-elevation acute coronary syndromes: insights from the Canadian Acute Coronary Syndrome Registries. Am Heart J. 2005;150(4):690-4. 10.1016/j.ahj.2004.11.012 16209967

[6] 6. Yan AT, Yan RT, Huynh T, DeYoung P, Weeks A, Fitchett DH, Langer A, Goodman SG; INTERACT Investigators. Bleeding and outcome in acute coronary syndrome: insights from continuous electrocardiogram monitoring in the Integrilin and Enoxaparin Randomized Assessment of Acute Coronary Syndrome Treatment (INTERACT) Trial. Am Heart J. 2008;156(4):769-75. 10.1016/j.ahj.2008.05.022 18926160

[7] 7. Gipson JS, Wood EM, Cole-Sinclair MF, McQuilten Z, Waters N, Woodford NW. Major haemorrhage fatalities in the Australian national coronial database. Emerg Med Australas. 2018;30(3):382-8. 10.1111/1742-6723.12915 29224237

[8] 8. Lozano R, Naghavi M, Foreman K, Lim S, Shibuya K, Aboyans V, et al. Global and regional mortality from 235 causes of death for 20 age groups in 1990 and 2010: a systematic analysis for the Global Burden of Disease Study 2010. Lancet. 2012;380(9859):2095-128. Erratum in: Lancet. 2013; 381(9867):628. AlMazroa, Mohammad A [added]; Memish, Ziad A [added]. 10.1016/S0140-6736(12)61728-0 PMC10790329 23245604

[9] 9. Cannon JW. Hemorrhagic Shock. N Engl J Med. 2018;378(4):370-9. Review. 10.1056/NEJMra1705649 29365303

[10] 10. Hunt BJ. Bleeding and coagulopathies in critical care. N Engl J Med. 2014; 370(22):847-59. 10.1056/NEJMc1403768 24869733

[11] 11. Gaunt C, Woolley T. Management of haemorrhage in major trauma. BJA Education. 2014;14(6):251-5.

[12] 12. Eastridge BJ, Holcomb JB, Shackelford S. Outcomes of traumatic hemorrhagic shock and the epidemiology of preventable death from injury. Transfusion. 2019;59(S2):1423-8. Review. 10.1111/trf.15161 30980749

[13] 13. Rossaint R, Bouillon B, Cerny V, Coats TJ, Duranteau J, Fernández-Mondéjar E, Filipescu D, Hunt BJ, Komadina R, Maegele M, Nardi G, Neugebauer E, Ozier Y, Riddez L, Schultz A, Vincent JL, Spahn DR; STOP Bleeding Campaign. The STOP the Bleeding Campaign. Crit Care. 2013;17(2):136. Review. 10.1186/cc12579 PMC3672629 23635083

[14] 14. Lee BY, Hong SB. Rapid response systems in Korea. Acute Crit Care. 2019;34(2):108-16. Review. 10.4266/acc.2019.00535 PMC6786673 31723915

