# Supplementary material for: Bleeding management after implementation of the Hemorrhage Code (Code H) at the Hospital Israelita Albert Einstein, São Paulo, Brazil
Source: Einstein (Sao Paulo). 2020 Aug 25;18:eAO5032. doi: 10.31744/einstein_journal/2020AO5032 (PMC9586425; doi:10.31744/einstein_journal/2020AO5032)
Supplement: Translation

# Translation: Manejo de sangramento após implantação do Código Hemorrágico (Código H) no Hospital Israelita Albert Einstein, São Paulo, Brasil

**Authors:** Michele Jaures, Neila Maria Marques Negrini Pigatti, Roseny dos Reis Rodrigues, Fernanda Paulino Fernandes, João Carlos de Campos Guerra

**Affiliations:** 1 Brasil Hospital Israelita Albert Einstein, São Paulo, SP, Brasil.

**Keywords:** Choque hemorrágico, Hemorragia, Transfusão de sangue, Transtornos da coagulação sanguínea

## Abstract

**Objetivo:**

Descrever a implantação de um protocolo de atendimento nos moldes de times de resposta rápida, para manejo e resolução do sangramento.

**Métodos:**

Foi idealizado e desenvolvido um protocolo institucional, chamado Código Hemorrágico, ou Código H, no qual, em um fluxo, um time multiprofissional e multidisciplinar foi constituído para atendimento integral, rápido e eficaz do paciente com doença hemorrágica grave. Em um outro fluxo, os profissionais, centralizados na farmácia do hospital, focavam na identificação do paciente com risco de hemorragia, no sentido de evitar o evento. Vários profissionais e setores do hospital foram treinados, e cada um, com funções específicas, deveria oferecer garantia total de apoio e suporte às equipes médica e de enfermagem.

**Resultados:**

Após a implementação do protocolo, o número de eventos catastróficos relacionados à falha no manejo do sangramento reduziu significativamente.

**Conclusão:**

O Código H é um exemplo de projeto de medicina baseada em valor e de medicina de precisão, por meio de atendimento integral e multidisciplinar, além de testes *point of care* introduzidos na prática clínica, com otimização da segurança do paciente e da prática assistencial na instituição. Ainda, por meio dele, deve ser possível minimizar o risco de ações judiciais para o hospital e os médicos, além de racionalizar recursos, com benefícios para administradores e fontes pagadoras.

## INTRODUÇÃO

A hemorragia grave é importante causa de mortalidade e morbidade em vários contextos clínicos, incluindo trauma, cirurgia e obstetrícia.^(1-7)^Foram estimadas 1,9 milhão de mortes por ano no mundo associadas à hemorragia e ao choque hemorrágico.^(8)^O choque hemorrágico é caracterizado por perda de sangue severa, que leva à entrega inadequada de oxigênio no nível celular e resulta rapidamente em morte. A mediana de tempo desde seu início até a morte é de 2 horas.^(9)^

Os eventos hemorrágicos não fatais resultam em necessidade de terapias adicionais, prolongamento do tempo de internação, uso de agentes hemostáticos e suspensão de agentes antitrombóticos. Essa suspensão, por sua vez, pode impactar em desfecho negativo para pacientes com risco trombótico.^(1-6)^

No Hospital Israelita Albert Einstein (HIAE), de janeiro de 2013 a abril de 2016, o número de eventos adversos catastróficos relacionados à falha no manejo do sangramento foi de 29% (n=14) dentre os eventos adversos catastróficos totais da instituição (n=49) (Figura 1).

**Figura 1:** Eventos adversos catastróficos associados ao manejo inadequado do sangramento

A causa de uma hemorragia pode ser complexa, e sua gestão pode ficar limitada por conta de ferramentas de diagnóstico^(10)^ e protocolos clínicos redundantes. Um dos pontos-chave do tratamento bem-sucedido é o tempo de ação. Além das intervenções rápidas, é essencial a coordenação entre os diferentes serviços do ambiente hospitalar e que exista um gerenciamento preventivo de sangramento.^(7)^

Diversas abordagens de manejo de hemorragias foram descritas nas últimas décadas. As mais recentes preconizam o controle rápido do sangramento, o manejo precoce da coagulopatia, a manutenção da perfusão adequada e a minimização da resposta inflamatória.^(11)^

Durante a análise de causa raiz (ACR) dos eventos adversos catastróficos do HIAE, (Figura 2), observou-se que as falhas ocorreram devido à ausência de reconhecimento precoce do sangramento, às falhas no tratamento, e à falta de logística e comunicação entre as áreas de apoio hospitalar envolvidas no diagnóstico e no tratamento desse paciente, culminando no atraso do diagnóstico e do atendimento do paciente.

**Figura 2:** Descrição dos eventos adversos catastróficos por falha no manejo do sangramento de janeiro 2013 a abril de 2016 EAG: evento adverso grave.

Em 2004, o *Institute for Healthcare Improvement* (IHI), por meio da campanha *The 100.000 Lives,* recomendou a implantação de times de resposta rápida (TRR) nos hospitais como uma das seis estratégias para reduzir a ocorrência de mortes inesperadas. Este TRR corresponde a um grupo de profissionais com expertise em cuidados críticos/intensivos, que deve ser rapidamente acionado à beira do leito do paciente.

Frente a estratégia dos TRR, surgiu a hipótese de que a aplicação de um protocolo multidisciplinar pudesse evitar os eventos adversos relacionados ao insucesso no manejo e no reconhecimento do choque hemorrágico.

## OBJETIVO

Descrever a implantação de um protocolo de atendimento nos moldes de times de resposta rápida, para manejo e resolução do sangramento.

## MÉTODOS

Relato de experiência retrospectivo de ações para implantação do Código Hemorrágico, ou Código H, no período de maio de 2016 a junho de 2019, um projeto de medicina baseada em valor e medicina de precisão. Nele, por meio de um atendimento integral e multidisciplinar, e de testes *Point of Care* introduzidos na prática clínica, a segurança do paciente e a prática assistencial obtêm melhorias.

O Código H tem como principais objetivos a identificação precoce de sinais e sintomas de sangramento, e a implementação rápida do tratamento, por meio de serviços de emergência institucionais. Este protocolo exige uma equipe bem coordenada de diferentes departamentos do hospital, como unidade de terapia intensiva (UTI), anestesia, banco de sangue, laboratório clínico, imagem diagnóstica, intervenção vascular e centro cirúrgico (Figura 3).

**Figura 3:** Fluxo de atendimento do Código Hemorrágico (Código H) * Paciente de trauma: deve ser acionada a equipe titular do trauma. CC: centro cirúrgico; PAS: pressão arterial sistólica; PAD: pressão arterial diastólica; FC: frequência cardíaca; CMC: clínica médica cirúrgica; RAVA: radiologia vascular ; UTI: unidade de terapia intensiva; USG: ultrassonografia; TC: tomografia computadorizada; Na: sódio; K: potássio; Ca: cálcio; TEG: tromboelastograma.

Os parâmetros utilizados para desencadear o Código H, também chamados como “pontos de acionamento”, ou “*trigger points*”, estão descritos no tabela 1.

**Tabela 1:** Pontos de acionamento

1. Presença de pressão arterial sistólica ≤90mmHg
2. Pressão arterial média-diastólica ≤60mmHg
3. Ritmo cardíaco ≥110bpm
4. Palidez cutânea da mucosa associada a exames de imagem que apontam para “sangramento ativo”
5. Hematoma ou coleta sugestiva de sangramento em cavidades
6. Resultado de “pânico” em testes de coagulação

Os indicadores do processo e do desfecho com relação às ações antes e após o Código H foram registrados entre janeiro 2013 e junho de 2019. As mensurações e análises se focalizaram nas seguintes métricas: número de eventos adversos relacionados ao manejo inadequado do sangramento e número de acionamentos do Código H, a partir de maio de 2016.

Os aspectos éticos desse relato foram respeitados, por se tratar de um levantamento retrospectivo de dados em prontuários, o que não interferiu no cuidado recebido pelo paciente. O presente estudo dispensou o Termo de Consentimento Livre e Esclarecido.

Seguindo as recomendações internacionais de manejo de sangramento e por meio de análise multidisciplinar dos eventos adversos catastróficos, um plano de ação foi desenhado, no intuito de garantir o controle rápido do sangramento, o manejo precoce da coagulopatia, a manutenção da perfusão adequada e a oferta dos serviços adequados em tempo hábil, de acordo com a etiologia do sangramento.

Assim, uma vez identificado um ou mais critérios dos pontos de acionamento, o Código H é ativado por meio da ligação para um número específico no local de atendimento do paciente. Automaticamente, todos os serviços relacionados recebem a notificação da chamada (laboratório, banco de sangue, centro cirúrgico, radiologia intervencionista, serviço de imagem, UTI, endoscopia, cirurgião do pronto atendimento, serviço de transporte e equipe de anestesia institucional) e têm um tempo de ação preconizado pelo protocolo (Figura 3).

A partir desse momento, deve ser realizada uma série de ações, sequenciais e paralelas, por diferentes profissionais de saúde (Figura 3):

- Os profissionais de enfermagem devem iniciar ou manter medidas de aquecimento do paciente, interromper medicações que potencializem o sangramento (como antiagregante, anticoagulante e trombolítico) e quantificar o sangramento. Isso auxilia na decisão médica de abertura de protocolos transfusionais.
- O profissional médico é responsável por decidir iniciar ou não transfusão de hemocomponentes imediatamente, e gerar ações que visam à manutenção da normotermia e à correção precoce dos níveis séricos de cálcio e do pH fisiológico. Além disso, deve identificar se a causa do choque hemorrágico é de etiologia cirúrgica ou de coagulopatia, para direcionar o paciente ao local de resolução do sangramento o quanto antes.
- Laboratório: precisa garantir que a coleta de exames seja realizada em até 5 minutos após a ativação do Código H, e a liberação dos resultados ocorra nos 20 minutos subsequentes. A coleta do pacote do Código H inclui hemograma completo com plaquetas, gasometria arterial com lactato, coagulograma com dosagem de fibrinogênio sérico, cálcio iônico, coágulo-check com razão normalizada internacional (RNI) à beira do leito, tromboelastometria e testes de avaliação da função plaquetária.
- Banco de sangue: é responsável por prover o transporte de dois concentrados de hemácias tipo O negativo dentro dos 20 primeiros minutos do acionamento. Caso o médico responsável pelo acionamento dispare o protocolo de transfusão maciça, o banco de sangue é responsável por manter o fluxo de fornecimento dos hemocomponentes, conforme necessidade de solicitação do caso.

Os serviços para resolução cirúrgica ou clínica do sangramento são acionados para disponibilizar leito, conforme a necessidade indicada pelo médico.

## RESULTADOS

A implantação de um protocolo multidisciplinar demandou, em primeiro lugar, o treinamento continuado de todos os membros envolvidos. O treinamento das equipes, o perfil e a atuação dos profissionais foram os principais fatores de sucesso para o resultado positivo do código H. Estes profissionais demonstraram ser dotados de habilidades técnicas e ter visão sistêmica, consciência situacional e práticas resolutivas, para reconhecimento e manejo do sangramento. No estágio inicial, cerca de 530 funcionários foram treinados, o que correspondeu a mais de 1.100 horas de treinamento.

Desde a implantação até junho de 2019, o Código H foi acionado 227 vezes (Figura 4). Observamos subutilização do código no início de sua implantação, acionamento excessivo em médio prazo e posterior adequação do acionamento nos períodos subsequentes.

**Figura 4:** Número de ativações do Código H no período de maio 2016 até junho de 2019

Uma das causas mais importantes para o não acionamento do Código H estava apoiada na percepção do médico responsável pelo paciente sobre sua capacidade de manejar o estado hemorrágico, sem a necessidade de outras equipes de apoio e suporte. O treinamento continuado e o apoio incondicional da instituição para empoderamento das equipes foram determinantes para que houvesse a mudança da cultura e o crescimento no volume de acionamentos.

A instituição trabalhava com modelos de atendimento por TRR desde 2005 com amplo sucesso, e a criação do código de manejo do sangramento embasado nesta estrutura trouxe familiaridade às equipes e rápida disseminação da facilidade dos serviços disponibilizados com o acionamento. Antes do modelo proposto, as diversas necessidades, como reservas de bolsas de sangue, sala de cirurgia ou exames, eram realizadas de forma individual e não sequenciada, ocasionando atrasos no atendimento do paciente.

Após a implantação do Código H, em maio de 2016, até junho de 2019, foi observada diminuição significativa do número de eventos adversos catastróficos relacionados à falha no manejo do sangramento (apenas um caso em 2017), conforme demonstrado na figura 5.

**Figura 5:** Evolutivo dos eventos adversos catastróficos totais e relacionados a choque hemorrágico antes e após a implantação do Código Hemorrágico (Código H) EAG: evento advrso grave; CH: choque hemorrágico.

## DISCUSSÃO

A hemorragia como consequência de procedimentos cirúrgicos ou devido a distúrbios de coagulação ocorre dentro da estatística de complicações de alguns procedimentos.^(1-7)^ Intervenções dedicadas ao controle do sangramento e ressuscitação hemostática têm demonstrado mérito na diminuição da mortalidade por injúria hemorrágica.^(12)^

Na campanha *Stop the Bleed* foram descritos como elementos essenciais para o controle do sangramento: avaliação de pacientes com risco de sangramento coagulopático; tratamento imediato do sangramento e coagulopatia, assim que eles se desenvolvam; observação contínua da resposta às intervenções; e desenvolvimento de estratégias no intuito de evitar sangramento secundário e coagulopatia.^(13)^

A ACR é uma das ferramentas de gestão de riscos amplamente utilizada na instituição. No presente trabalho, associou-se a ACR com a expertise dos TRR na elaboração de um protocolo multidisciplinar e sequenciado para manejo de sangramento. Desde sua criação nos anos 2000, os TRR têm sido aplicados em diversas partes do mundo e em diversos casos clínicos, com amplo sucesso.^(14)^

O Código H acrescenta ainda como elementos importantes o tempo de resposta, a alocação do paciente no local correto, e a coordenação multidisciplinar e multisetorial.

Importante ressaltar o aspecto positivo na formação das equipes multidisciplinares advindo do treinamento para a implantação do Código H. A base de informações e as diretrizes proporcionadas às equipes trouxeram maior envolvimento e percepção dos sinais de sangramento pelas equipes multidisciplinares. Efeitos significativos deste treinamento foram demonstrados no aumento gradual do número de acionamentos, e na condução da resolução do sangramento, proporcionando um desfecho positivo para os pacientes atendidos. A ausência de reconhecimento dos sinais de sangramento era um dos pontos frágeis identificados em falhas no atendimento, e está descrito em diversas literaturas como ponto-chave na manutenção da vida do paciente. Foi possível incorporar um programa de treinamento reprodutível e sustentável para as equipes que capilarizam o conhecimento sobre o sangramento e capacitam novos funcionários, além de reforçar as habilidades das equipes.

O número de eventos adversos catastróficos na instituição reduziu consideravelmente após a implantação do protocolo, o que ratifica a importância deste tipo de iniciativa para a melhoria contínua dos serviços de saúde.

Existem certas limitações deste estudo, principalmente no custo financeiro da implantação do Código H. Estudos adicionais estão sendo realizados, com o intuito de avaliar o impacto desta iniciativa na gestão hospitalar.

## CONCLUSÃO

A implantação de um protocolo institucional, com investimentos em treinamento de equipe médica e multidisciplinar, reduziu o número de eventos adversos relacionados a sangramento dentro da instituição. A sistematização do atendimento permitiu reduzir as falhas relacionadas ao atendimento desta intercorrência. Isso gerou impacto na redução da morbimortalidade e, dessa forma, concluímos que o Código H contribui positivamente para a qualidade e a segurança da assistência prestada ao paciente com sangramento.

O Código H é um exemplo de projeto de medicina baseada em valor e medicina de precisão, no qual, por meio de um atendimento integral e multidisciplinar, e de testes *Point of Care* introduzidos na prática clínica, melhoramos a segurança do paciente e a prática assistencial em nossa instituição. Como resultados adicionais, deve ser possível minimizar o risco de ações judiciais para o hospital e os médicos, além de racionalizar recursos com benefícios para administradores e fontes pagadoras.
